# **The association between hyperuricemia and insulin resistance surrogates, dietary- and lifestyle insulin resistance indices in an Iranian population: MASHAD cohort study**

**DOI:** 10.1186/s12937-023-00904-2

**Published:** 2024-01-04

**Authors:** Najmeh Seifi, Mina Nosrati, Glareh Koochackpoor, Malihe Aghasizadeh, Hossein Bahari, Hedyeh Beheshti Namdar, Nafiseh Afkhami, Reza Assaran Darban, Farnoosh Azarian, Gordon A. Ferns, Majid Ghayour-Mobarhan

**Affiliations:** 1 International UNESCO Center for Health-Related Basic Sciences and Human Nutrition, Mashhad University of Medical Sciences, Mashhad, Iran; 2 School of Nursing and Allied Medical Sciences, Maragheh University of Medical Sciences, Maragheh, Iran; 3 Department of Nutrition, Faculty of Medicine, Mashhad University of Medical Sciences, Mashhad, Iran; 4 Department of Nutrition Sciences, Varastegan Institute for Medical Sciences, Mashhad, Iran; 5 Department of Biology, Faculty of Sciences, Mashhad Branch, Islamic Azad University, Mashhad, Iran; 6 Division of Medical Education, Sussex Medical School, Brighton &amp, Falmer, Brighton, Sussex UK; 7 International UNESCO Center for Health-Related Basic Sciences and Human Nutrition, Mashhad University of Medical Sciences, Mashhad, Iran 9919991766

**Keywords:** Hyperuricemia, Insulin resistance, Empirical dietary index for insulin resistance, Empirical lifestyle index for insulin resistance

## Abstract

**Background:**

Previous studies have reported insulin resistance (IR) to be associated with hyperuricemia. In this study, we aimed to assess the possible associations between the empirical dietary index for IR (EDIR), the empirical lifestyle index for IR (ELIR), and non-insulin-based surrogates (triglyceride–glucose (TyG) index, triglyceride-to-high-density-lipoprotein-cholesterol (TG/HDL-C) ratio, metabolic score for insulin resistance (METS-IR) and TyG with body mass index (TyG-BMI)) and hyperuricemia in an Iranian population.

**Methods:**

In this cross-sectional study, 6457 participants aged 35–65 years were recruited as part of the MASHAD cohort study. EDIR and ELIR were calculated using dietary intakes, body mass index, and physical activity information. Insulin resistance surrogates including TyG, TyG-BMI, TG/HDL-C, and METS-IR were calculated for all participants. Hyperuricemia was defined as serum uric acid ≥ 7 mg/dl in men or ≥ 6 mg/dl in women. Multivariable logistic regression models were applied to determine the association between indexes of IR and hyperuricemia.

**Results:**

The mean ELIR and IR surrogates (TyG, TyG-BMI, TG/ HDL, and METS-IR) were significantly higher in subjects with hyperuricemia compared to non-hyperuricemic subjects (p < 0.001). After adjusting for confounding variables, the association between hyperuricemia and EDIR was not significant, but ELIR had a significant association in all models (p < 0.001). All four IR surrogates (TyG, TyG-BMI, TG/ HDL, and METS-IR) showed a significant association with hyperuricemia (p < 0.001).

**Conclusion:**

There was a significant association between indexes of insulin resistance: TyG, TyG-BMI, TG/HDL-c, METS-IR, and ELIR with hyperuricemia, in a population sample from northeastern Iran.

## Background

Hyperuricemia, usually defined as serum uric acid concentration > 7 mg/dl [1], is estimated to occur in approximately 8.9–24.4% of the general population [2]. The clinical consequence of hyperuricemia includes gouty arthritis and chronic musculoskeletal pains; however, several studies have reported that even asymptomatic hyperuricemia increases the risk of a wide range of cardiometabolic disorders, including resistant hypertension, hyperlipidemia, metabolic syndrome, type II diabetes, chronic kidney disease, cardiovascular events, and ischemic cardiac diseases [3].

The association between insulin resistance (IR) and hyperuricemia has been shown in previous studies, suggesting that insulin resistance may lead to hyperuricemia [4–6]. Some other studies have shown that hyperuricemia is associated with peripheral insulin resistance and this may partially mediate the effect of hyperuricemia on the development of metabolic disorders [7, 8]. Although the role of insulin resistance, whether as a cause or as a mediator that aggravates the consequences of hyperuricemia, has been supported by some studies. The assessment of insulin resistance using methods that rely on plasma insulin levels is invasive, expensive and difficult in large cohort studies, and not applicable in clinical screening [8]. Therefore, there have been efforts to determine surrogate measures of insulin resistance that are less complex, non-invasive, and less expensive.

Non-insulin-based indexes of IR, empirical dietary index for IR (EDIR), and empirical lifestyle index for IR (ELIR) have been proposed as alternatives to the traditional indicators of IR [9]. The association between hyperuricemia and non-insulin-based indexes of IR has been assessed in a limited number studies that have been inconsistent in their results. Although lifestyle and dietary habits are known as the most important modifiable determinants of IR, to our knowledge, no study has investigated the association between hyperuricemia with EDIR and ELIR, so the present study aims to assess the association of six indexes of IR, including TyG, TG/HDLc, TyG-body mass index (BMI), METS-IR, EDIR and ELIR with hyperuricemia in an Iranian population.

## Methods

### Study settings and population

Participants of this cross-sectional study were recruited as part of the Mashhad Stroke and Heart Atherosclerotic Disorder (MASHAD) cohort study. The inclusion criteria were 35–65 years old and living in Mashhad city. A total of 9704 participants were originally recruited [10]. Demographic data, anthropometric measurements, dietary intake, physical activity level (PAL), and laboratory measurements were determined at baseline [10]. Individuals with incomplete dietary intake data, women who were pregnant or breastfeeding, and those without serum acid uric data were excluded (n = 3247). Finally, 6457 individuals remained for the final analysis. All individuals provided written informed consent, and the study was performed in accordance with the declaration of Helsinki, and was approved by the Human Research Ethics Committee of Mashhad University of Medical Sciences (IR.MUMS.MEDICAL.REC.1398.228).

### Dietary intake assessment

The frequency of consuming 65 food items was reported daily, weekly or monthly and never/seldom responded for the preceding year. The frequency of consumption of each food item was converted into a daily basis. The portion sizes were defined as the reference serving sizes and converted into grams based on household measures [11]. The FFQ was completed by face-to-face interviews with experienced nutritionist. A total of 6457 FFQs were completed. We used Diet Plan 6 software (Forestfield Software Ltd., Horsham, West Sussex, UK) in order to calculate macro and micronutrients intake, including carbohydrates, protein, fat (saturated, unsaturated, and trans fats), and food items were categorized into red meat, high fat dairy, refined grain, and fruit.

### Physical activity assessment

The physical activity questions based on the James and Schofield equation were selected from the Scottish Heart Health Study (SHHS)/ MONICA questionnaire [12]. Questions were divided into time spent on activities during work, non-work time, and in bed. The integrated energy index (IEI) for women at inactive, moderate, and active levels were 1.61, 2.52, and 4.39, respectively. The IEI for men at inactive, moderate, and active levels were 1.51, 2.49, and 4.34, respectively. Considering time spent on each activity and the IEI physical activity was calculated.

### Laboratory data and definitions

Blood samples (20 ml) were collected from each participant after a 14 h overnight fasting into plain tubes. All blood samples were centrifuged at room temperature to separate the plasma and serum [10]. Triglyceride (TG), total cholesterol (TC), high-density lipoprotein cholesterol (HDL-C), low-density lipoprotein cholesterol (LDL-C) and uric acid were determined using enzymatic methods on an BT 3000 automated analyzer, by commercial kits (Pars Azmoon, Iran). According to the extant guidelines, the cut-off values for lipid profile are TG ≥ 1.7 mmol/L (150 mg/dl), TC ≥ 5.0 mmol/L (200 mg/dl), LDLC ≥ 3.4 mmol/L (130 mg/dl), HDL-C < 1.3 mmol/L (50 mg/dl) (for women) and HDL-C < 1.04 mmol/L (40 mg/dl) (for men) [13]. Hyperuricemia was defined as uric acid (UA) ≥ 420 mol/L (7 mg/dl) for men or UA ≥ 360 mol/L (6 mg/dl) for women [14].

Insulin Resistance (IR) substitutes were TyG-BMI, TyG index, TG/HDL-C, and METS-IR, calculated using the following formulae [15]:

- TyG = ln [TG (mg/dl) * FPG (mg/dl) / 2]
- TyG-BMI: = TyG index * BMI
- TG/HDL-C = TG (mg/dl) / HDL-C (mg/dl);
- METS-IR = (ln [2 * FPG (mg/dl) + TG (mg/dl)] * BMI (kg/m2) / ln [HDL-C (mg/dl)]).

### EDIR and ELIR indexes

The Empirical Dietary Insulin Resistance (EDIR) and Empirical Lifestyle Insulin Resistance (ELIR) indexes were reported in previous studies [16].

The EDIR score contains 12 items, including fish, red meat, processed meats, refined grains, tomatoes, other vegetables, and fruit juice, which are positively associated with IR; and green leafy vegetables, dark yellow vegetables, coffee, nuts, and high-fat dairy products which are negative determinants. Also, the ELIR score included both dietary and lifestyle items, including Body Mass Index (BMI), physical activity and two groups of food components, including positive (red meat, refined grains, low-energy beverages, tomatoes, fruit juice, potatoes, processed meat, other vegetables, and tea) and negative (coffee, green and leafy vegetables, and high-fat dairy) determinants. Dietary items were changed into serving sizes per 1000 Kcal energy expenditure. Each item (BMI, physical activity (MET.h/wk.), and dietary components) was multiplied with their weights, and then values for all items of EDIR and ELIR were summed to attain their final score [17]. The higher EDIR and ELIR index scores indicate the potential role of diet and lifestyle items in elevating the risk of insulin resistance [17].

### Statistical analysis

We classified participants based on the presence of hyperuricemia as defined using the cut-off mentioned above. General characteristics of study participants were presented as means ± standard deviation (SDs) for continuous variables and frequency number (percentages) for categorical variables. We used an independent t-test for continuous variables and the chi-square test for categorical variables to examine the differences between the two groups. Then, to assess the association between metabolic syndrome and EDIR, ELIR, TyG, TyG-BMI, TG/HDL-c, and METS-IR scores, we classified participants based on quartile cut-off points of the scores and used multivariable logistic regression to estimate odd ratios (ORs) and 95% CIs in crude and multivariable-adjusted models. In the first model, we adjusted for age and sex. Additional adjustments were made for BMI (except for ELIR), energy intake, education level, smoking status, and physical activity level (except for ELIR). In the full adjustment model, we further adjusted for glomerular filtration rate and history of chronic diseases, including diabetes, hypertension, and dyslipidemia. To obtain the overall trend of ORs across quartiles of scores, we considered these quartiles as continuous variables. All statistical analyses were done using the Statistical Package for Social Sciences (version 25; SPSS Inc.). P < 0.05 was considered statistically significant.

## Results

Six thousand four hundred fifty-seven individuals were included in the final analysis. Table 1 shows the characteristics of participants separated into hyperuricemia and non-hyperuricemia groups. A total of 558 (8.6%) subjects had hyperuricemia. The hyperuricemia group were significantly older, more likely to be men, having higher BMI and waist circumference, and lower physical activity level (p < 0.05). Systolic and diastolic blood pressures were also significantly higher in the hyperuricemia group (p < 0.001). The hyperuricemia group also had higher EDIR and ELIR scores (p = 0.02 and p < 0.001, respectively). The mean serum uric acid and TG concentrations and IR surrogates (TyG, TyG-BMI, TG/ HDL, and METS-IR) were also significantly higher in the hyperuricemia group than in the other group (p < 0.001).

**Table 1:** General demographic, clinical and laboratory characteristics of individuals with and without hyperuricemia

	With hyperuricemia N = 558	Without Hyperuricemia N = 5899	p-value
Age (y)	50.73 ± 8.13^*^	48.22 ± 8.17	<0.001
Gender Male, n (%)	244 (43.72) ^**^	2335(39.58)	0.03
Education University educated n (%)	38(6.8)	392(6.6)	0.46
BMI (kg/m^2^)	30.15 ± 4.63	27.78 ± 4.68	<0.001
Waist circumference (cm)	99.31 ± 11.36	94.46 ± 12.15	<0.001
SBP (mmHg)	128.27 ± 20.84	121.82 ± 18.31	<0.001
DBP (mmHg)	82.66 ± 11.83	79.24 ± 10.95	<0.001
PAL	13.29 ± 2.46	13.81 ± 2.51	<0.001
History of chronic disease n (%) CVD Diabetes HTN Dyslipidemia	85(15.2) 65(11.6) 192(34.4) 190(34)	533(9) 687(11.6) 1055(17.9) 1271(21.5)	< 0.001 0.51 < 0.001 < 0.001
Serum uric acid (mol/L)	439.20 ± 92.61	264.93 ± 65.46	<0.001
LDL cholesterol (mg/dl)	119.10 ± 41.66	116.14 ± 34.65	0.10
HDL cholesterol (mg/dl)	42.44 ± 10.30	43.21 ± 9.98	0.08
Triglycerides (mg/dl)	199.89 ± 128.41	137.10 ± 83.23	<0.001
EDIR	0.37 ± 0.34	0.41 ± 0.42	0.02
ELIR	1.76 ± 0.32	1.69 ± 0.38	<0.001
TyG	8.98 ± 0.59	8.56 ± 0.64	< 0.001
TyG-BMI	271.34 ± 46.74	238.72 ± 47.50	< 0.001
TG-HDL ratio	5.15 ± 3.98	3.46 ± 2.59	<0.001
METS-IR	48.19 ± 8.43	42.80 ± 8.71	<0.001
TyG	8.98 ± 0.59	8.56 ± 0.64	<0.001
TyG-BMI	271.34 ± 46.74	238.72 ± 47.50	<0.001
TG-HDL ratio	5.15 ± 3.98	3.46 ± 2.59	<0.001
METS-IR	48.19 ± 8.43	42.80 ± 8.71	<0.001

BMI: body mass index; CVD: cardiovascular disease; DBP: diastolic blood pressure; EDIR: empirical dietary index for insulin resistance; ELIR: empirical lifestyle index for insulin resistance; HDL: high density lipoprotein; LDL: HTN: hypertension; low density lipoprotein; METS-IR: metabolic score for insulin resistance; PAL: physical activity level; SBP: systolic blood pressure; TG/HDLc: triglyceride to high-density lipoprotein cholesterol ratio; TyG: the product of fasting triglycerides and glucose. ^*^Represented as mean ± SD, independent t-test was applied ^**^represented as number (percent), Chi-square analysis was applied

As shown in Table 2, carbohydrate and refined grain intakes were higher in the non-hyperuricemia than in the hyperuricemia group (p = 0.04 and p < 0.001, respectively). High-fat dairy intake was significantly higher in the hyperuricemia group (p < 0.001). Intakes of protein and red meat were also higher in the hyperuricemia group, but these were not statistically significant.

**Table 2:** Nutritional intakes of individuals with and without hyperuricemia

	With hyperuricemia N = 558	Without Hyperuricemia N = 5899	p-value
Energy (kcal)	1958.26 ± 588.68 ^*^	1997.62 ± 591.21	0.13
Carbohydrate (g/day)	275.35 ± 86.92	286.05 ± 122.88	0.04
Total sugar (g/ day)	129.55 ± 50.95	131.28 ± 53.55	0.47
Fiber (g/day)	25.53 ± 9.92	26.01 ± 9.92	0.28
Protein (g/day)	76.23 ± 22.70	75.31 ± 22.50	0.36
Fat (g/day)	66.58 ± 28.65	66.68 ± 26.84	0.93
MUFA (g/day)	27.71 ± 22.25	27.41 ± 21.32	0.76
PUFA (g/day)	8.57 ± 3.21	8.58 ± 3.08	0.97
Saturated fat (g/day)	29.35 ± 13.71	29.64 ± 12.92	0.62
Refined grain (serving/day)	2.25 ± 2.99	2.68 ± 3.78	< 0.001
Red meat (serving/day)	0.33 ± 0.30	0.32 ± 0.28	0.30
High-fat dairy (serving/day)	1.26 ± 0.69	1.18 ± 0.61	< 0.001
Fruits (serving/day)	1.68 ± 1.40	1.56 ± 1.41	0.06

MUFA: mono unsaturated fatty acid; PUFA: poly unsaturated fatty acid. Independent t-test was used to compare variables in the two groups. * represented as mean ± SD.

Table 3 shows a multivariate logistic regression analysis for the association of hyperuricemia with EDIR and ELIR indexes. We indicated odds ratio (OR) and 95% confidence interval (95% CI) for the highest versus the lowest quartile. According to this table, after adjusting variables in all models, the association between hyperuricemia and EDIR was not significant, but ELIR showed a significant association in all models (p < 0.001).

**Table 3:** Multiple logistic regression analysis models for the association between hyperuricemia (dependent variable) and EDIR and ELIR (independent variables)

	EDIR quartiles					ELIR quartiles				
	Q1	Q2	Q3	Q4	p-trend	Q1	Q2	Q3	Q4	p-trend
Model 1	1	0.95 (0.74–1.22) ^*^	0.98 (0.77–1.26)	1.02 (0.80–1.31)	0.83	1	1.73 (1.31–2.36)	2.43 (1.83–3.22)	2.79 (2.10–3.70)	< 0.001
Model 2	1	0.94 (0.73–1.22)	0.98 (0.76–1.28)	1.07 (0.82–1.42)	0.57	1	1.79 (1.34–2.41)	2.52 (1.90–3.36)	3.13 (2.34–4.18)	< 0.001
Model 3	1	0.86 (0.62–1.19)	0.79 (0.57–1.10)	0.88 (0.63–1.22)	0.38	1	1.32 (0.92–1.91)	1.66 (1.16–2.38)	1.93 (1.33–2.80)	< 0.001

Model1: adjusted for age, sex; Model2: Model1 + BMI (only for EDIR), energy intake, education level, smoking status, physical activity level (only for EDIR); Model3: Model2 + chronic diseases including diabetes, hypertension, or dyslipidemia, and estimated glomerular filtration rate. BMI: body mass index; EDIR: empirical dietary index for insulin resistance; ELIR: empirical lifestyle index for insulin resistance. Multiple logistic regression analysis was applied. ^*^shows odds ratio (confidence interval)

Table 4 indicates the relationship between hyperuricemia and four IR surrogates (TyG, TyG-BMI, TG/ HDL, and METS-IR) by multi-variable logistic regression. The results showed that after adjusting variables in all models, there was a significant association between hyperuricemia and all IR surrogates.

**Table 4:** Multiple logistic regression analysis models for the association between hyperuricemia (dependent variable) and insulin resistance surrogates (independent variables)

Variable	TyG quartiles					TyG-BMI quartiles				
	Q1	Q2	Q3	Q4	p-trend	Q1	Q2	Q3	Q4	p-trend
Model 1	1	2.95 (1.94–4.40) ^*^	5.66 (3.85–8.32)	8.68 (5.96–12.65)	< 0.001	1	2.47 (1.70–3.59)	4.32 (3.03–4.15)	8.17 (5.79–11.52)	< 0.001
Model 2	1	2.45 (1.68–3.86)	4.49 (3.03–6.67)	6.81 (4.62–10.04)	< 0.001	1	2.52 (1.69–3.76)	4.51 (2.94–6.84)	8.53 (5.17–14.05)	< 0.001
Model 3	1	3.34 (1.91–5.87)	5.59 (3.25–9.61)	10.04 (5.85–17.24)	< 0.001	1	3.27 (1.95–5.51)	5.70 (3.25-10.00)	14.49 (7.33–28.63)	< 0.001
**Variable**	**TG/HDL quartiles**					**METS-IR quartiles**				
	**Q1**	**Q2**	**Q3**	**Q4**	**p-trend**	**Q1**	**Q2**	**Q3**	**Q4**	**p-trend**
Model 1	1	2.03 (1.42–2.89)	3.48 (2.50–4.84)	5.65 (4.11–7.77)	< 0.001	1	2.11 (1.48–3.01)	3.31 (02.37–4.63)	6.42 (4.66–8.84)	< 0.001
Model 2	1	1.81 (1.26–2.60)	2.98 (2.12–4.19)	4.65 (3.34–6.49)	< 0.001	1	1.95 (1.34–2.85)	2.94 (1.98–4.35)	5.27 (3.33–8.34)	< 0.001
Model 3	1	2.36 (1.44–3.86)	3.59 (2.24–5.74)	6.08 (3.82–9.68)	< 0.001	1	2.02 (1.25–3.28)	3.35 (2.05–5.56)	6.64 (3.64–12.11)	< 0.001

Model1: adjusted for age, and sex; Model2: Model1 + BMI, energy intake, education level, smoking status, physical activity level; Model3: Model2 + chronic diseases including diabetes, hypertension, or dyslipidemia, and estimated glomerular filtration rate. TG/HDLc: triglyceride to high-density lipoprotein cholesterol ratio; TyG: the product of fasting triglycerides and glucose; BMI: body mass index; METS-IR: metabolic score for insulin resistance. Multiple logistic regression analysis was applied. ^*^Shows odds ratio (confidence interval)

## Discussion

In this cross-sectional study, we investigated the associations of two different surrogate measures of insulin resistance, including: non-insulin-based indexes (TG/ HDL, TyG, TyG-BMI, and METS-IR) and empirical indexes (EDIR and ELIR) with odds of having hyperuricemia. We found that four non-insulin-based indexes of IR were significantly associated with the risk of hyperuricemia. Among these four indexes, TyG-BMI had the strongest association with hyperuricemia; the participants in the highest quartile of TyG-BMI had 14 times greater risk of hyperuricemia than participants in the lowest quartile. Our findings also indicate that a lifestyle associated with a higher ELIR score might be related to increased hyperuricemia risk. However, this was not the case for EDIR. To the best of our knowledge, this study has for the first time, simultaneously investigated the relationship between six simple indexes of IR and hyperuricemia risk in an Iranian adult population.

Insulin resistance may take several years to evolve into type 2 diabetes [18]. However, several studies have shown that until the onset of diabetes, IR could be a contributing factor in several pathological conditions [19]. Since the methods used to estimate IR in many of these studies are invasive, impractical fro large scale studies, not accurate, and less reproducible, a series of subsequent studies investigated the efficacy of insulin resistance surrogate markers in estimating the risk of IR-related disease and observed a strong association between these indexes with cardiometabolic diseases [20, 21] chronic kidney disease [22] and hypertension [15]. However, few studies have investigated the association between non-insulin-based indexes and hyperuricemia. In our study, increasing the score of all non-insulin-based indexes of IR was associated with an increase in the risk of hyperuricemia. In another study by Liu and his colleagues, the associations between hyperuricemia and three non-insulin-based IR indexes, including TG/HDLc, TyG, and METS-IR, were investigated. In this study, increased TG/HDLc and TyG scores were associated with an increased risk of hyperuricemia, but no significant relationship between hyperuricemia and METS-IR score was observed [23]. In explaining this discrepancy between the two studies on the relationship between METS-IR score and risk of hyperuricemia, it should be noted that the mean score of METS-IR in our study was higher than reported by Liu et al. (48.19 ± 8.43 vs. 33.1 ± 9.5). In the study of Han et al. [24], the cutoff of METS-IR, which is a predictor of hyperuricemia in patients with type 2 diabetes, was reported as 46.33 (sensitivity: 57.9%, specificity: 71.0%), which is very close to the mean score of METS-IR in our study. However, the cutoff reported in Han et al.‘s study is in diabetic patients; therefore, more studies are needed to find the METS-IR cutoff associated with an increased risk of hyperuricemia in non-diabetic individuals.

In the current study, TyG-BMI was the most potent indicator of hyperuricemia. However, in the study by Han et al. [24], TyG/HDL-c was the best marker for identifying hyperuricemia. In addition to the difference in the study population, Han et al.‘s study was conducted on diabetic patients while our participants did not have diabetes; this contradiction may result from the BMI’s inability to distinguish between fat and muscle, especially for Asian individuals [25]. Previous studies have also shown a dose-response relationship between BMI and serum uric acid level [26]. Adiposity is associated with the accumulation of free fatty acids in the liver, increased triglyceride synthesis and an increased production of uric acid through the activation of the uric acid synthesis pathway [27].

Our study shows that a higher ELIR is significantly associated with the increased risk of hyperuricemia. The results of our study are in line with the findings of previous studies that reported the direct association of ELIR score with increasing the risk of various chronic diseases such as cardiometabolic diseases [17], coronary heart disease outcomes [28], and diabetes [29]. At the same time, we have not seen this association in the EDIR score. The ELIR index contains three items: food component, BMI level, and physical activity, while the EDIR score only includes food component, so it seems that the synergistic effect of three important determinants of the ELIR index (food component, high BMI level, and sedentary lifestyle) can intensify the insulinemic potential of diet in the progression of hyperuricemia. In addition, lifestyle is generally a stronger predictor of insulin response than diet alone [30].

Our study had several strengths, including the appropriate sample size to explore the relationship between our exposures (serum insulin resistance surrogates) and desired outcomes. Also, the present study is the first investigation that assessed the possible role of the Iranian dietary and lifestyle insulinemic potential indexes on the risk of hyperuricemia. However, there are some limitations of the present study. First, this cross-sectional observational study does not allow us to confer casual relationships between serum insulin resistance surrogates and odds of hyperuricemia. Second, the FFQ is known to be subject to reporting errors.

## Conclusion

There a significant association between TyG, TyG-BMI, TG/HDL-c, METS-IR, and ELIR with hyperuricemia in a population sample from the city of Mashhad, Iran, even though no significant association was observed between the EDIR score and odds of hyperuricemia.

## Data Availability

The datasets generated and/or analyzed during the current study are not publicly available due to university data ownership policies, but are available from the corresponding author on reasonable request.

## Abbreviations

BMI: body mass index
CVD: cardiovascular disease
DBP: diastolic blood pressure
EDIR: empirical dietary index for insulin resistance
ELIR: empirical lifestyle index for insulin resistance
FFQ: food-frequency questionnaire
HDL: high density lipoprotein
HTN: hypertension
LDL: low density lipoprotein
METS-IR: metabolic score for insulin resistance
PAL: physical activity level
SBP: systolic blood pressure
TG/HDLc: triglyceride to high-density lipoprotein cholesterol ratio
TyG: the product of fasting triglycerides and glucose BMI:body mass index

## References

[1] Dincer HE, Dincer AP, Levinson DJ. Asymptomatic hyperuricemia: to treat or not to treat. Cleve Clin J Med. 2002;69(8):594, 7, 600-2 passim. 10.3949/ccjm.69.8.594 12184468

[2] Perez-Ruiz F, Dalbeth N, Bardin T (2015). A review of uric acid, crystal deposition Disease, and gout. Adv Ther.

[3] Wang H, Zhang H, Sun L, Guo W (2018). Roles of hyperuricemia in metabolic syndrome and cardiac-kidney-vascular system Diseases. Am J Transl Res.

[4] Facchini F, Chen YD, Hollenbeck CB, Reaven GM (1991). Relationship between resistance to insulin-mediated glucose uptake, urinary uric acid clearance, and plasma uric acid concentration. JAMA.

[5] McCormick N, O’Connor MJ, Yokose C, Merriman TR, Mount DB, Leong A (2021). Assessing the Causal relationships between insulin resistance and hyperuricemia and gout using bidirectional mendelian randomization. Arthritis Rheumatol.

[6] Ter Maaten JC, Voorburg A, Heine RJ, Ter Wee PM, Donker AJ, Gans RO (1997). Renal handling of urate and sodium during acute physiological hyperinsulinaemia in healthy subjects. Clin Sci (Lond).

[7] Han T, Lan L, Qu R, Xu Q, Jiang R, Na L (2017). Temporal relationship between hyperuricemia and insulin resistance and its impact on future risk of Hypertension. Hypertension.

[8] Wardhana W, Rudijanto A (2018). Effect of uric acid on blood glucose levels. Acta Med Indones.

[9] Tabung FK, Wang W, Fung TT, Hu FB, Smith-Warner SA, Chavarro JE (2016). Development and validation of empirical indices to assess the insulinaemic potential of diet and lifestyle. Br J Nutr.

[10] Ghayour-Mobarhan M, Moohebati M, Esmaily H, Ebrahimi M, Parizadeh SMR, Heidari-Bakavoli AR (2015). Mashhad Stroke and heart atherosclerotic disorder (MASHAD) study: design, baseline characteristics and 10-year cardiovascular risk estimation. Int J Public Health.

[11] Ahmadnezhad M, Asadi Z, Miri HH, Ebrahimi-Mamaghani M, Ghayour-Mobarhan M, Ferns GA. Validation of a short semi-quantitative food frequency questionnaire for adults: a pilot study. J Nutritional Sci Dietetics. 2017:49–55.

[12] Bolton-Smith C, Woodward M, Tunstall-Pedoe H (1992). The Scottish Heart Health Study. Dietary intake by food frequency questionnaire and odds ratios for coronary Heart Disease risk. II. The antioxidant vitamins and fibre. Eur J Clin Nutr.

[13] Expert Panel on Detection E (2001). Executive summary of the third report of the National Cholesterol Education Program (NCEP) expert panel on detection, evaluation, and treatment of high blood cholesterol in adults (Adult Treatment Panel III). JAMA.

[14] Bardin T, Richette P (2014). Definition of hyperuricemia and gouty conditions. Curr Opin Rheumatol.

[15] Li Y, You A, Tomlinson B, Yue L, Zhao K, Fan H (2021). Insulin resistance surrogates predict Hypertension plus hyperuricemia. J Diabetes Invest.

[16] Mokhtari E, Teymoori F, Farhadnejad H, Mirmiran P, Azizi F (2022). Development and validation of dietary and lifestyle insulinemic indices among Iranian adult population. Nutr Metabolism.

[17] Teymoori F, Jahromi MK, Ahmadirad H, Daftari G, Mokhtari E, Farhadnejad H (2023). The association of dietary and lifestyle indices for insulin resistance with the risk of cardiometabolic Diseases among Iranian adults. Sci Rep.

[18] Di Pino A, DeFronzo RA (2019). Insulin resistance and Atherosclerosis: implications for insulin-sensitizing agents. Endocr Rev.

[19] Lebovitz HE (2001). Insulin resistance: definition and consequences. Exp Clin Endocrinol Diabetes.

[20] Rojas-Humpire R, Olarte-Durand M, Medina-Ramirez S, Gutierrez-Ajalcriña R, Canaza JF, Huancahuire-Vega S (2021). Insulin resistance indexes as biomarkers of Lifetime Cardiovascular risk among adults from Peru. J Nutr Metab.

[21] Wang B, Zhang M, Liu Y, Sun X, Zhang L, Wang C (2018). Utility of three novel insulin resistance-related lipid indices for predicting type 2 Diabetes Mellitus among people with normal fasting glucose in rural China. J Diabetes.

[22] Shen FC, Lin HY, Tsai WC, Kuo IC, Chen YK, Chao YL (2023). Non-insulin-based insulin resistance indices for predicting all-cause mortality and renal outcomes in patients with stage 1–4 chronic Kidney Disease: another paradox. Front Nutr.

[23] Liu XZ, Xu X, Zhu JQ, Zhao DB (2019). Association between three non-insulin-based indexes of insulin resistance and hyperuricemia. Clin Rheumatol.

[24] Han R, Zhang Y, Jiang X (2022). Relationship between four non-insulin-based indexes of insulin resistance and serum uric acid in patients with type 2 Diabetes: a cross-sectional study. Diabetes Metab Syndr Obes.

[25] Zierle-Ghosh A, Jan A, Physiology. Body Mass Index. StatPearls. Treasure Island (FL): StatPearls Publishing Copyright © 2023. StatPearls Publishing LLC.; 2023. 30571077

[26] Yang L, He Z, Gu X, Cheng H, Li L. Dose–response relationship between BMI and Hyperuricemia. Int J Gen Med. 2021:8065–71. 10.2147/IJGM.S341622 PMC8594780 34795514

[27] Matsuura F, Yamashita S, Nakamura T, Nishida M, Nozaki S, Funahashi T (1998). Effect of visceral fat accumulation on uric acid metabolism in male obese subjects: visceral fat obesity is linked more closely to overproduction of uric acid than subcutaneous fat obesity. Metabolism.

[28] Teymoori F, Mokhtari E, Farhadnejad H, Mirmiran P, Rad HA, Azizi F (2022). The dietary and lifestyle indices of insulin resistance are associated with increased risk of Cardiovascular Diseases: a prospective study among an Iranian adult population. Nutr Metab Cardiovasc Dis.

[29] Farhadnejad H, Mokhtari E, Teymoori F, Sohouli MH, Moslehi N, Mirmiran P (2021). Association of the insulinemic potential of diet and lifestyle with risk of Diabetes incident in Tehranian adults: a population based cohort study. Nutr J.

[30] Yang W, Sui J, Zhao L, Ma Y, Tabung FK, Simon TG (2021). Association of Inflammatory and Insulinemic Potential of Diet and Lifestyle with risk of Hepatocellular Carcinoma. Cancer Epidemiol Biomarkers Prev.

